# Supplementary figures and images for: Public parks and the pandemic: How park usage has been affected by COVID-19 policies
Source: PLoS One. 2021 May 19;16(5):e0251799. doi: 10.1371/journal.pone.0251799 (PMC8133454; doi:10.1371/journal.pone.0251799)

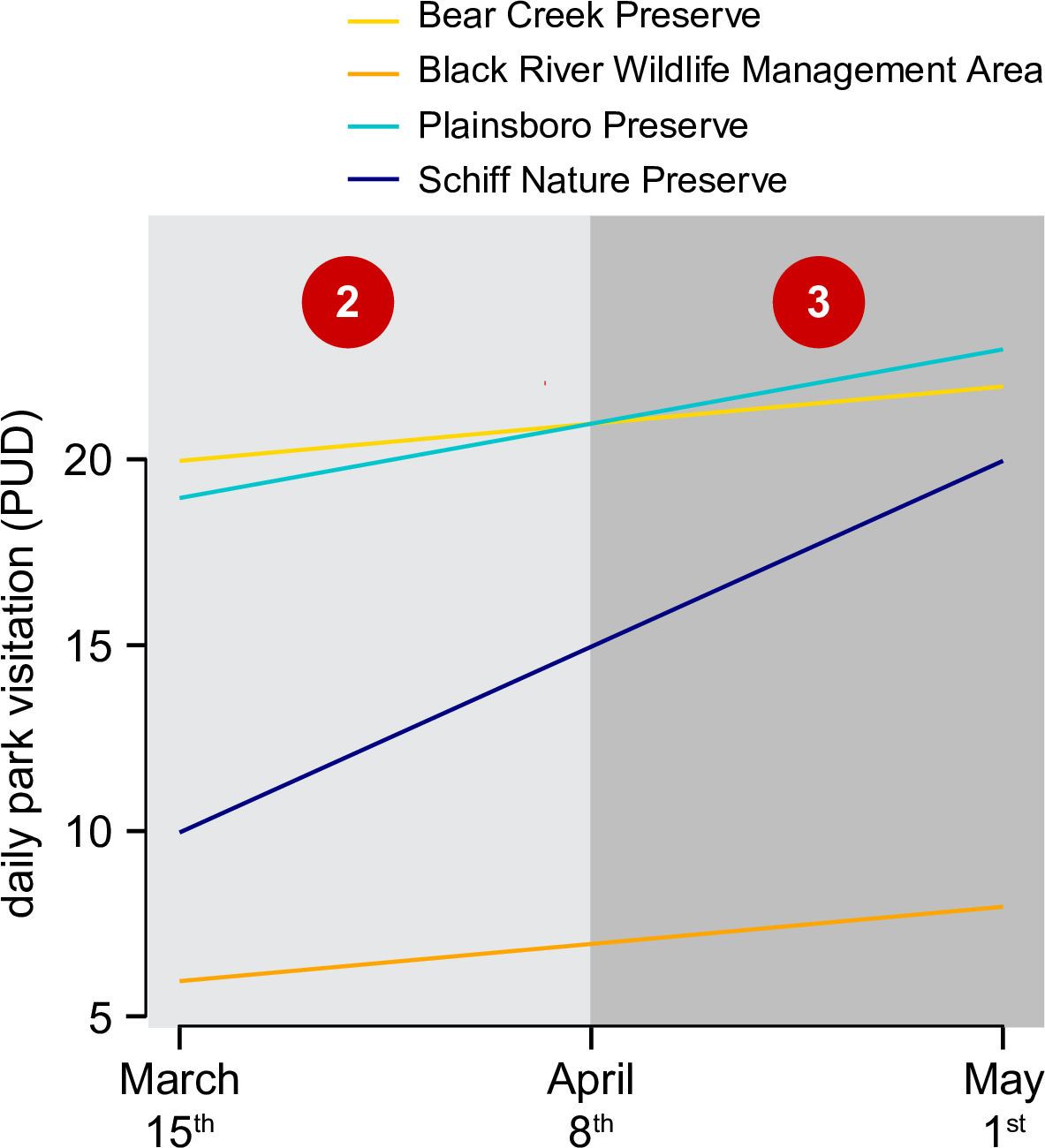

Supplement: S1 Fig — Visitation to Schiff Nature Preserve almost doubled, whereas increases were much more moderate in Bear Creek Preserve, Black River Wildlife Management Area, and Plainsboro Preserve. The numbers in red circles refer to the time periods denoted in Fig 1. (TIF) [file pone.0251799.s001.tif]

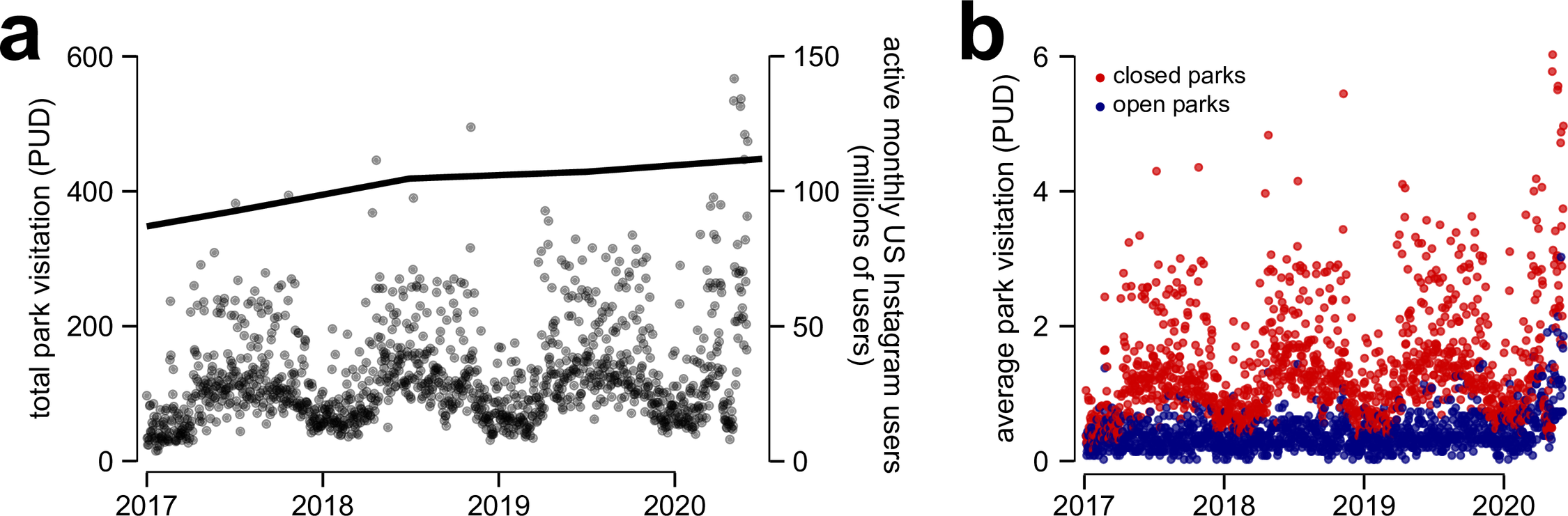

Supplement: S2 Fig — (a) Total park visitation (PUD summed across all parks) during the study period (2017–2020) as well as (b) average visitation for parks that were closed or open during the executive order. The black line in (a) reflects the trend in the number of active US Instagram users through time, which was used to detrend PUD values when comparing park usage across years. Note in (a) that Instagram usage has increased through time, and also the distinct seasonality of park visitation. Likewise, note in (b) that parks that were closed during the park shutdown consistently had higher visitation historically than parks that remained open. (TIF) [file pone.0251799.s002.tif]
