# Supplementary material for: Public parks and the pandemic: How park usage has been affected by COVID-19 policies
Source: PLoS One. 2021 May 19;16(5):e0251799. doi: 10.1371/journal.pone.0251799 (PMC8133454; doi:10.1371/journal.pone.0251799)
Supplement: S1 File — (DOCX) [file pone.0251799.s003.docx]

**List of sources consulted to determine park closure status during the shutdown**

1. Friends of Hopewell Valley Open Space. *COVID-19 Updates.* 2020. [cited April 18th, 2020]. Available at: *https://www.fohvos.info/covid-19-updates/*
2. Hunterdon County Division of Parks and Recreation. *Your County Parks*. 2020. [cited April 18th, 2020]. Available at: *http://www.co.hunterdon.nj.us/depts/parks/guides/parkareas .htm*
3. New Jersey Department of Environmental Protection. *Open spaces at state parks and wildlife management areas available for passive recreation; enclosed facilities, campgrounds at state parks, forests and recreation areas closed*. 2020. [cited April 18th, 2020]. Available at: *https://www.nj.gov/dep/newsrel/2020/20_0010.htm*
4. NJ.com. *What N.J. parks are closed? A full list of parks Gov. Murphy just closed in response to coronavirus.* 2020**.** [cited April 18th, 2020]. Available at: *https://www.nj.com/ coronavirus/2020/04/is-my-local-park-closed-a-full-list-of-parks-gov-murphy-just-closed-in-response-to-coronavirus.html*
5. Planet Princeton. *Open and closed: Princeton area parks.* 2020. [cited April 18th, 2020]. Available at: *https://planetprinceton.com/2020/04/08/open-and-closed-princeton-area-parks/*
6. Somerset County Parks Division. *COVID-19 Press Release.* 2020. [cited April 18th, 2020]. Available at: *https://www.somersetcountyparks.org/COVID-19%20Press%20Release %209-2.pdf*
7. Tewksbury Township. *COVID-19 Resources.* 2020. [cited April 18th, 2020]. Available at: *https://www.tewksburytwp.net/coronavirus_(covid-19)/coronavirus_(covid-19).php*
8. Union County. *Parks, Activities, Locations & Maps.* 2020**.** [cited April 18th, 2020]. Available at: *https://ucnj.org/parks-activities/*
9. Village Green NJ. *Essex County Closes All Parks, Playgrounds, Dog Parks to Prevent Spread of COVID-19.* 2020. [cited April 18th, 2020]. Available at: *https://villagegreennj.com/ towns/government/march-16-2020-essex-county-announces-additional-precautions-to-prevent-spread-of-covid-19/*
