# Supplementary material for: Public parks and the pandemic: How park usage has been affected by COVID-19 policies
Source: PLoS One. 2021 May 19;16(5):e0251799. doi: 10.1371/journal.pone.0251799 (PMC8133454; doi:10.1371/journal.pone.0251799)
Supplement: Attachment — Submitted filename: PONE-D-20-35168_reviewer_12_16_2020.pdf [file pone.0251799.s004.pdf]

PLOS ONE

Public parks and the pandemic: how park usage has been affected by COVID-19 policies

--Manuscript Draft--

**Manuscript Number:**	PONE-D-20-35168
**Article Type:**	Research Article
**Full Title:**	Public parks and the pandemic: how park usage has been affected by COVID-19 policies
**Short Title:**	The use of public parks during the COVID-19 pandemic
**Corresponding Author:**	Joel Opila Abraham<br>Princeton University<br>Princeton, NJ UNITED STATES
**Keywords:**	COVID-19; Instagram; New Jersey; Mental Health; pandemic; public parks; recreation
**Abstract:**	Public parks serve an important societal function as recreational spaces for diverse communities of people, with well documented physical and mental health benefits. As such, parks may be crucial for how people have handled effects of the COVID-19 pandemic, including increasingly limited recreational opportunities, widespread financial uncertainty, and consequent heightened anxiety. However, despite the documented benefits of parks, many states have instituted park shutdown orders due to fears that public parks could facilitate COVID-19 transmission. Here we use geotagged social media data from New Jersey parks to examine (1) whether park visitation increased when the COVID-19 pandemic began and (2) whether park shutdown orders are effective at deterring park usage. We find that park visitation increased substantially with the onset of the pandemic. The subsequent park shutdown order in New Jersey caused visitation in closed parks to decline significantly while parks that remained open mostly continued to experience elevated visitation. Visitation then returned to elevated pre-shutdown levels when closed parks were allowed to reopen. Altogether, our results indicate that parks continue to provide crucial services to society, particularly in stressful times during which opportunities for recreation are limited. Furthermore, our results suggest that policies targeting changes in human behavior can be effective and are also largely reversible. As such, we should continue to invest in public parks and to explore the role of parks in managing public health and psychological well-being.
**Order of Authors:**	Zoe Merrill Volenec
	Joel Opila Abraham
	Alex David Becker
	Andrew P Dobson
**Additional Information:**	
**Question**	**Response**
**Financial Disclosure**<br><br>Enter a financial disclosure statement that describes the sources of funding for the work included in this submission. Review the submission guidelines for detailed requirements. View published research articles from *PLOS ONE* for specific examples.<br><br>This statement is required for submission	The authors received no specific funding for this work.

and **will appear in the published article** if the submission is accepted. Please make sure it is accurate.<br><br>**Unfunded studies**<br>Enter: *The author(s) received no specific funding for this work.*<br><br>**Funded studies**<br>Enter a statement with the following details:<br>• Initials of the authors who received each award<br>• Grant numbers awarded to each author<br>• The full name of each funder<br>• URL of each funder website<br>• Did the sponsors or funders play any role in the study design, data collection and analysis, decision to publish, or preparation of the manuscript?<br>• **NO** - Include this sentence at the end of your statement: *The funders had no role in study design, data collection and analysis, decision to publish, or preparation of the manuscript.*<br>• **YES** - Specify the role(s) played.<br><br>* typeset	
**Competing Interests**<br><br>Use the instructions below to enter a competing interest statement for this submission. On behalf of all authors, disclose any competing interests that could be perceived to bias this work—acknowledging all financial support and any other relevant financial or non-financial competing interests.<br><br>This statement **will appear in the published article** if the submission is accepted. Please make sure it is accurate. View published research articles from *PLOS ONE* for specific examples.	The authors have declared that no competing interests exist.

**NO authors have competing interests**<br><br>Enter: *The authors have declared that no competing interests exist.*<br><br>**Authors with competing interests**<br><br>Enter competing interest details beginning with this statement:<br><br>*I have read the journal's policy and the authors of this manuscript have the following competing interests: [insert competing interests here]*<br><br>* typeset	
**Ethics Statement**<br><br>Enter an ethics statement for this submission. This statement is required if the study involved:<br><br>• Human participants<br>• Human specimens or tissue<br>• Vertebrate animals or cephalopods<br>• Vertebrate embryos or tissues<br>• Field research<br><br>Write "N/A" if the submission does not require an ethics statement.<br><br>General guidance is provided below. Consult the submission guidelines for detailed instructions. **Make sure that all information entered here is included in the Methods section of the manuscript.**	N/A

**Format for specific study types**<br><br>**Human Subject Research (involving human participants and/or tissue)**<br>• Give the name of the institutional review board or ethics committee that approved the study<br>• Include the approval number and/or a statement indicating approval of this research<br>• Indicate the form of consent obtained (written/oral) or the reason that consent was not obtained (e.g. the data were analyzed anonymously)<br><br>**Animal Research (involving vertebrate animals, embryos or tissues)**<br>• Provide the name of the Institutional Animal Care and Use Committee (IACUC) or other relevant ethics board that reviewed the study protocol, and indicate whether they approved this research or granted a formal waiver of ethical approval<br>• Include an approval number if one was obtained<br>• If the study involved *non-human primates*, add *additional details* about animal welfare and steps taken to ameliorate suffering<br>• If anesthesia, euthanasia, or any kind of animal sacrifice is part of the study, include briefly which substances and/or methods were applied<br><br>**Field Research**<br><br>Include the following details if this study involves the collection of plant, animal, or other materials from a natural setting:<br>• Field permit number<br>• Name of the institution or relevant body that granted permission	
**Data Availability**<br><br>Authors are required to make all data underlying the findings described fully available, without restriction, and from the time of publication. PLOS allows rare exceptions to address legal and ethical concerns. See the PLOS Data Policy and FAQ for detailed information.	Yes - all data are fully available without restriction

A Data Availability Statement describing where the data can be found is required at submission. Your answers to this question constitute the Data Availability Statement and **will be published in the article**, if accepted.<br><br>**Important:** Stating 'data available on request from the author' is not sufficient. If your data are only available upon request, select 'No' for the first question and explain your exceptional situation in the text box.<br><br>Do the authors confirm that all data underlying the findings described in their manuscript are fully available without restriction?	
**Describe where the data may be found in full sentences. If you are copying our sample text, replace any instances of XXX with the appropriate details.**<br><br>• If the data are **held or will be held in a public repository**, include URLs, accession numbers or DOIs. If this information will only be available after acceptance, indicate this by ticking the box below. For example: *All XXX files are available from the XXX database (accession number(s) XXX, XXX.*).<br>• If the data are all contained **within the manuscript and/or Supporting Information files**, enter the following: *All relevant data are within the manuscript and its Supporting Information files.*<br>• If neither of these applies but you are able to provide **details of access elsewhere**, with or without limitations, please do so. For example:<br><br>*Data cannot be shared publicly because of [XXX]. Data are available from the XXX Institutional Data Access / Ethics Committee (contact via XXX) for researchers who meet the criteria for access to confidential data.*<br><br>*The data underlying the results presented in the study are available from (include the name of the third party*	All relevant data will be available from the Dryad data repository (accession number TBD).

and contact information or URL).<br>• This text is appropriate if the data are owned by a third party and authors do not have permission to share the data.<br><br>* typeset	
Additional data availability information:	Tick here if the URLs/accession numbers/DOIs will be available only after acceptance of the manuscript for publication so that we can ensure their inclusion before publication.

<u>FULL TITLE:</u> Public parks and the pandemic: how park usage has been affected by COVID-19 policies

SHORT TITLE: The use of public parks during the COVID-19 pandemic

AUTHORS: Zoe Volenec[1*], Joel Abraham[1*†], Alexander D. Becker[2], and Andy Dobson[1]

AFFILIATIONS:

[1]Department of Ecology and Evolutionary Biology, Princeton University, Princeton, NJ, 08544

[2]Department of Biology, Stanford University, Stanford, CA, 94305

*These authors contributed equally to this work.

†Correspondence: email: joeloa@princeton.edu, phone: +1 (609) 258-5810, address: Department of Ecology and Evolutionary Biology, Princeton University, Princeton, NJ, 08544

KEYWORDS: COVID-19; Instagram; New Jersey; mental health; pandemic; public parks; recreation

Figures, tables, textboxes: 5 figures, 0 tables, 0 textboxes

Length of abstract: 231 words

Length of main text: 5853 words

Number of citations: 48

ABSTRACT

Public parks serve an important societal function as recreational spaces for diverse communities of people, with well documented physical and mental health benefits. As such, parks may be crucial for how people have handled effects of the COVID-19 pandemic, including increasingly limited recreational opportunities, widespread financial uncertainty, and consequent heightened anxiety. However, despite the documented benefits of parks, many states have instituted park shutdown orders due to fears that public parks could facilitate COVID-19 transmission. Here we use geotagged social media data from New Jersey parks to exa[illegible]e (1) whether park visitation increased when the COVID-19 pandemic began and (2) whether park shutdown orders are effective at deterring park usage. We find that park visitation increased substantially with the onset of the pandemic. The subsequent park shutdown order in New Jersey caused visitation in closed parks to decline significantly while parks that remained open mostly continued to experience elevated visitation. Visitation then returned to elevated pre-shutdown levels when closed parks were allowed to reopen. Altogether, our results indicate that parks continue to provide crucial services to society, particularly in stressful times during which opportunities for recreation are limited. Furthermore, our results suggest that policies targeting changes in human behavior can be effective and are also largely reversible. As such, we should continue to invest in public parks and to explore the role of parks in managing public health and psychological well-being.

INTRODUCTION

The ongoing COVID-19 pandemic has disrupted many aspects of human social life, limiting recreational opportunities and affecting peoples' financial security (Freeman and Eykelbosh 2020; Goldberg *et al.* 2020). Likewise, many schools have been forced to close due to COVID-19, creating the need for parents to educate and entertain their children at home (Viner *et al.* 2020). These disruptions have resulted in profound increases in stress, anxiety, and depression (Jacobson *et al.* 2020; Torales *et al.* 2020). As a result, people have had to seek out alternative means of recreation and of managing their mental and physical health, along with that of their children (Honey-Roses *et al.* 2020; Jacobson *et al.* 2020; Viner *et al.* 2020).

Public parks have always served a critical function as free recreational spaces (Ulrich and Addoms 1981; Godbey *et al.* 1992; Manning and More 2002), particularly for low income and immigrant communities (Jennings *et al.* 2012; Wolch *et al.* 2014; Anguelovski 2020). Parks and green spaces more generally are known to have positive impacts on mental health (Ulrich and Addoms 1981), with demonstrated mental health benefits (Nutsford *et al.* 2013; Wood *et al.* 2017; Buckley *et al.* 2019). Also, parks can be quite expansive, allowing for safe enjoyment while maintaining social distancing (Freeman and Eykelbosh 2020). As such, pa[illegible] may serve an important – and as of yet unquantified – function for people in coping with the COVID-19 pandemic (Honey-Roses *et al.* 2020; Samuelsson *et al.* 2020; Venter *et al.* 2020).

The possible appeal of parks has made them the focus of policy attention in the United States during the ongoing pandemic (CDC 2020; Ellis 2020; Korevaar *et al.* 2020). Because of their function as recreational spaces for diverse communities, policymakers have worried that parks could serve as transmission hotspots for COVID-19 (Ellis 2020; Honey-Roses *et al.* 2020; Freeman and Eykelbosh 2020; Venter *et al.* 2020). Parks, especially those tailored towards

children, have many shared surfaces, which has been hypothesized to result in increased disease transmission (CDC 2020; Freeman and Eykelbosh 2020). Furthermore, parks are often unsupervised and lack guidelines for visitor capacity, making it difficult to regulate their usage (CDC 2020; Freeman and Eykelbosh 2020).

In response to these concerns, some states have instituted park shutdowns, entirely preventing people from using open spaces (Ellis 2020; Korevaar *et al.* 2020). These park shutdowns have been somewhat criticized for cutting people off from the benefits of publicly available outdoor areas (Freeman and Eykelbosh 2020; Honey-Roses *et al.* 2020), which disproportionately impacts lower income individuals with limited access to green spaces (Anguelovski 2020; Du *et al.* 2020; Valentino-DeVries *et al.* 2020). That said, it is unclear how effective these park shutdown orders have been at reducing public park usage and thereby achieving their purpose of diminishing transmission risk. On one hand executive orders shutting down parks may indeed act as a deterrent, but alternatively people may knowingly or unknowingly ignore the park shutdown orders. Attempts to discern the effects of these park closure policies have been limited, hindering the ability of policy makers to effectively evaluate their implementation (Honey-Roses *et al.* 2020).

Determining the efficacy of park shutdown orders requires case studies of states that have instituted such shutdowns. New Jersey was at the forefront of the United States COVID-19 outbreak, experiencing large outbreaks relatively early in the pandemic (Ellis 2020; Korevaar *et al.* 2020; Liu *et al.* 2020). As such, the state had to lead the charge in crafting and instituting policies to respond to the pandemic, including an executive order by New Jersey's governor Governor Murphy shutting down state [illegible] county parks (Ellis 2020; Higgins-Dunn and Kim 2020; Korevaar *et al.* 2020). This executive park shutdown order did not go into effect until

nearly a month after stay-at-home restrictions were put in place and was subsequently lifted about a month later (Fig. 1; Higgins-Dunn and Kim 2020). Thus, New Jersey provides an important case study for understanding both (1) the immediate impacts of COVID-19 on the use of public parks and (2) the efficacy and reversibility (*i.e.* the ability to return to pre-order conditions) of policies like the park shutdown order.

Here, we assess visitation in parks throughout New Jersey using geotagged Instagram photography data from February 2017 through May 2020 (Fig. 2; Wood *et al.* 2013; Donahue *et al.* 2017; Tenkanen *et al.* 2017; Hamstead *et al.* 2018). With this data we compare park usage during four periods of the pandemic: before the pandemic began (period 1); during the beginning of the pandemic (period 2), after the stay-at-home order was instituted but before the park shutdown; during the governor's park shutdown order (period 3); and following the lifting of the shutdown (period 4), when parks were allowed to reopen (Fig. 1). We evaluate three questions. First, how did park usage in New Jersey change with the onset of the COVID-19 pandemic? Second, how did the New Jersey governor's executive order to shut down parks affect the use of public parks? And finally, how did the lifting of the park shutdown order impact subsequent park visitation? We predict that (1) [illegible]k usage increased significantly with the start of the pandemic, given the crucial role that parks play as fee-free recreational spaces; (2) the executive order significantly decreased park visitation to closed parks, but parks that remained open saw higher visitation as people congregated in the remaining open parks; and (3) the withdrawal of the executive order caused park visitation to increase, possibly exceeding visitation before the closure as people compensated for lack of access to parks (Fig. 1).

RESULTS

Role of public parks in the pandemic

As hypothesized, we found that the onset of the pandemic was associated with increases in park usage. Park visitation in 2020 was higher during the first month of quarantine than during the same time frame in previous years, even when accounting for increased Instagram usage over time and variations in weather (Fig. 3a). Likewise, we observed that park visitation was higher in the ~3.5 weeks following COVID-19 quarantine restrictions (period 2, before park closures) than in the preceding 3.5 weeks of 2020 (period 1, before the pandemic), exceeding rates of temperature increase (Fig. 3b). Together, these findings demonstrate that the onset of the pandemic was associated with increased utiliz[illegible] of public parks.

Efficacy of the executive order

We found that the executive order to shut down parks was associated with decreased visitation to state and county parks, suggesting it was largely effective at restricting park usage. While closed parks saw significant declines in visitation from pre-order levels (Fig. 4b), not all parks that remained open saw predicted increases in visitation from pre-order levels and some even saw slight declines (Fig. 4a). In fact, only four of the 12 open parks analyzed here saw increases in visitation following the executive order (Fig. S1), which contrasts notably with our hypothesis that people would concentrate in the parks that remained open (Fig. 1). On whole, parks that remained open after the executive order experienced less of a decline, if any, in visitation relative to those that were closed during the shutdown (Fig. 4a).

Lifting of the executive order

Finally, we found that park usage largely returned to pre-executive order levels (period 2) following the lifting of the shutdown order (period 4). Park usage was higher after the executive order was lifted than when the shutdown order was in effect; both parks that were closed by the executive order and those that remained open saw increases in visitation in May (period 4) compared to the park shutdown period (period 3), though parks that were closed by the executive order experienced larger increases in visitation in May than those that had remained open (Fig. 5b). However, park usage following park reopening was no higher than pre-executive order levels on whole. Visitation in May (period 4) did not differ from visitation in the mid-March/early April quarantine period before parks were closed (period 2), for both closed and open parks (Fig. 5c). Likewise, park usage following the order was still higher than in previous years; park visitation in May, aggregated across all parks, was higher in 2020 as compared to visitation during May of the previous three years (Fig. 5a). In fact, of the ten most popular days for park visitation during our study period (2017-2020), seven were in May 2020 (Fig. S2a). Thus, the lifting of the shutdown order was associated with increases in park visitation to elevated pre-order levels.

DISCUSSION

By using social media data as a proxy for park visitation, we found (1) that park usage increased substantially with the onset of the pandemic. After the New Jersey governor issued an executive order to close parks, (2) park usage declined substantially and consistently in closed parks, whereas parks that remained open saw variable changes in visitation. Curiously, park usage increased in only a small fraction of open parks (4 of 12) following the executive order, contrary to our predictions (Fig. 1). Finally, with park reopening, (3) we found that park usage returned to, but did not exceed, elevated visitation levels before the governor's executive order.

Role of public parks in the pandemic

First and foremost, we observed an increase in public park use with the onset of the COVID-19 pandemic, as measured by posts on social media. This increase indicates that people relied more on public parks during the beginning of the pandemic for the diverse services that parks provide (Ulrich and Addoms 1981; Godbey *et al.* 1992; Manning and More 2002; Freeman and Eykelbosh 2020; Honey-Roses *et al.* 2020; Venter *et al.* 2020; Deforche *et al.* 2020). Most immediately, people probably visited parks more during the beginning of quarantine due to their accessibility: many public parks are free recreational spaces that are accessible (at least in theory) to diverse communities of people (Ulrich and Addoms 1981; Godbey *et al.* 1992; Manning and More 2002; Anguelovski 2020). These aspects of parks make them particularly appealing in the face of the widespread financial uncertainty and decreased mobility of quarantine (Freeman and Eykelbosh 2020; Goldberg *et al.* 2020; Rundle *et al.* 2020). In addition, with COVID-19-related school closures, many parents have been left to engage and educate their children (Viner *et al.* 2020). As such, parks have likely served as an outlet for families with

children in particular, enabling families to get out of the house and entertain their kids during the pandemic (Rundle *et al.* 2020; Matias *et al.* 2020; Viner *et al.* 2020). Finally, parks are known to have positive impacts on mental health (U[illegible]n and Addoms 1981; Nutsford *et al.* 2013; Wood *et al.* 2017; Buckley *et al.* 2019). Therefore, some of the observed increases in park usage during the initial stages of quarantine were likely driven by heightened anxiety and depression as the pandemic became more serious (Jacobson *et al.* 2020; Torales *et al.* 2020). Our findings are consistent with other work demonstrating that people increasingly made use of outdoor spaces for recreation during the pandemic. A study in Oslo, Norway that used mobile phone tracking data found that outdoor recreational activity increased during lockdown (Venter [illegible]*l.* 2020). Altogether, such findings emphasize that people have continued to rely on parks during the pandemic for the important and dynamic services that they provide.

The observed increases in park visitation offer an optimistic outlook for peoples' mental and physical health during quarantine. Some have suggested that quarantine restrictions might have ancillary health consequences due to decreased opportunities for recreation and physical activity (Rundle *et al.* 2020). Our results suggest that such concerns may be somewhat overexaggerated (Matias *et al.* 2020; Venter *et al.* 2020). Observed increases in the use of outdoor spaces may compensate for overall declines in mobility, thereby enabling people to maintain both their physical and mental well-being (Deforche *et al.* 2020; Jacobson *et al.* 2020; Matias *et al.* 2020). Though we do not have the data to investigate this possibility here, future research should evaluate whether increased use of parks has indeed been able to compensate for other reductions in mobility and physical activity with lockdown.

While public parks are in theory accessible to everyone, in reality the distribution of publicly available outdoor spaces in New Jersey disproportionately benefits predominantly

affluent and predominantly white communities (Wolch *et al.* 2014; Anguelovski 2020). Communities composed mainly of people of color resultantly have less access to parks and outdoor recreational spaces (and other public infrastructure more generally), despite increased reliance on such spaces for the services they provide (Jennings *et al.* 2012; Wolch *et al.* 2014; Anguelovski 2020). A notable aspect of the COVID-19 pandemic in the United States has been its disproportionate impact on these marginalized communities, particularly Black, Hispanic, and Native American communities (Fortuna *et al.* 2020; Haynes *et al.* 2020). Our results suggest that the lack of access to parks and other public infrastructure may contribute to the disparities in COVID-19 burden observed in these communities, as others have speculated (Fortuna *et al.* 2020; Haynes *et al.* 2020). By being unable to access parks, these communities are barred from the aforementioned physical and mental health benefits associated with parks at a time when these services are particularly critical (Jennings *et al.* 2012; Wolch *et al.* 2014; Samuelsson *et al.* 2020). As such, increasing the accessibility and equitability of public infrastructure such as parks may be crucial for curbing this and future pandemics (Du *et al.* 2020; Fortuna *et al.* 2020; Haynes *et al.* 2020). To better understand this relationship, further research should focus on how access to public infrastructure relates to COVID-19 burden, particularly within marginalized communities.

Efficacy of the executive order

We also found here that people were highly responsive to the governor's executive order to close parks. We observed substantive declines in visitation to closed parks associated with the executive order, suggesting that the order was largely effective at deterring park visitation. Likewise, the executive order seemingly did not induce panic; in contrast to our predictions (Fig.

1), people did not concentrate in open parks, though a few open parks did see visitation increases (possibly reflecting some uncertainty as to which parks were open or closed). Altogether, this underscores that clear guidance and policies from governing bodies do influence peoples' behavior during moments of crisis (Goldberg *et al.* 2020). Indeed, this finding parallels other research emphasizing the importance of strong leadership and clear guidelines for managing the pandem[illegible]recent study found significant increases in mask wearing and mask buying after the CDC formally recommended that Americans wear face masks to prevent the spread of COVID-19 (Goldberg *et al.* 2020). As such, clear leadership and policies are crucial for influencing behavior and managing moments of crisis.

It is possible that other changes in behavior may contribute to the observed reductions in park visitation to closed parks. The park shutdown order was instituted during a peak in the number of new COVID-19 cases in New Jersey (Fig. 1). As such, people may have independently decided to avoid public parks because they were increasingly nervous to leave home as the seriousness of the situation became apparent. In addition, observed declines in park visitation with the shutdown order may reflect decreases in Instagram posting rather than declines in park usage itself. People may still have been visiting parks, but not posting photos for fear of social or legal repercussions. To further complicate matters, this effect could be counteracted by retrospective Instagram posts during the shutdown. While we removed Instagram photos posted retrospectively as best we could using caption data (see *M*[illegible]*ls and Materials*), retrospective pictures might have driven some of the observed park usage, particularly the usage we observed when parks were closed. However, because such photos would serve to inflate visitation during the shutdown, actual reductions in park usage are likely even greater than what we capture here. While we cannot directly evaluate the contribution of

these factors to observed patterns, our results suggest an awareness of the executive order regardless. The executive order clearly changed peoples' behaviors, even if not necessarily in the intended way. This result is reassuring, as it means that people clearly knew about the shutdown order. Thus, if the shutdown order had failed, it would not have been for lack of knowledge of the policy. As such, disseminating knowledge of policies such as this is not a major barrier to their effective implementation.

Though the park shutdown order seems to have been largely effective at reducing park usage, this does not mean that it was necessarily the best policy for curtailing the pandemic. Other work has suggested that parks are not the transmission centers for COVID-19 as some have supposed (Ellis 2020; Freeman and Eykelbosh 2020; Honey-Roses *et al.* 2020) and may instead even help to mitigate the spread of the virus (Venter *et al.* 2020). Indeed, we observe that reopening parks was not associated with increases in COVID-19 cases in New Jersey. In fact, new cases continued to decline even after public parks were reopened in New Jersey and park visitation went back up (Fig. 1), though other policies for preventing the spread of COVID-19 were also in place that had not been previously. Still, given such findings and the aforementioned benefits that parks serve, we suggest that park shutdowns may not be the best means of curbing the pandemic. Instead, we should continue to expand our public park infrastructure and increase the accessibility of existing parks so that more people can access the multifaceted benefits that parks provide, particularly in moments of crisis such as those created by the current COVID-19 pandemic.

Lifting of the executive order

While we observed that people largely stopped using parks in response to the park shutdown order, public park use resumed immediately once they reopened. Once the order was lifted, people visited parks at the same levels that they had been before the order. This suggests that peoples' relationships to parks are quite plastic, depending on social and recreational context, and that the observed increases in park visitation during the onset of the pandemic were driven largely by the paucity of other recreational options (Freeman and Eykelbosh 2020; Venter *et al.* 2020; Deforche *et al.* 2020). It remains to be seen whether we will see enduring effects of the pandemic on park visitation after quarantine is over (Honey-Roses *et al.* 2020). Once restrictions are lifted, many people will likely go back to their alternative forms of recreation (Deforche *et al.* 2020; Honey-Roses *et al.* 2020). However, others may discover that time spent in parks has many benefits that they had not previously appreciated.

This observation has more general implications for how people may behave once quarantine ends. Some have speculated that the pandemic will have lasting impacts on how people interact with public spaces (Deforche *et al.* 2020; Du *et al.* 2020; Honey-Roses *et al.* 2020). Our finding suggests that this may not be the case. We observed that people returned quite quickly to parks when they reopened, and that they visited parks at the same rates they had before the shutdown. This could also be the case with other forms of recreation as they reopen. Once restrictions are lifted, peoples' behavior may return to what it was before quarantine, and our results suggest that these behavioral changes could be quite immediate (Honey-Roses *et al.* 2020; Jacobson *et al.* 2020; Samuelsson *et al.* 2020). While on the one hand it is discouraging that certain behavioral changes may not persist after quarantine, this finding does serve to underscore the adaptability and resilience that people have exhibited in response to the pandemic (Honey-Roses *et al.* 2020; Jacobson *et al.* 2020).

Finally, our results suggest that the effects of the executive order were largely reversible; the lifting of the order did not cause people to visit parks any more than they had been before the order. This further emphasizes that people are highly responsive to such orders and policies, and also makes clear that policy decisions do not need to be thought of as final or irreversible. Policymakers and leaders can and should explore policy options in real time to evaluate which policies are most effective for dealing with the specific situation at hand. We are not beholden to the first response we try; there is space to experiment.

CONCLUSIONS

Our study has two clear takeaways. First, we found that public parks continue to serve a crucia[illegible]e as recreational spaces (Ulrich and Addoms 1981; Godbey *et al.* 1992; Manning and More 2002), particularly in circumstances where recreational outlets are limited (Honey-Roses *et al.* 2020; Samuelsson *et al.* 2020; Venter *et al.* 2020). Throughout the world, and particularly in New Jersey, the human population is expanding (Morse *et al.* 2012; Woods *et al.* 2007; Wu 2010), meaning that the demand for parks and outdoor recreational spaces will only increase. Given their well-documented benefits and ongoing functionality, we should therefore continue to invest in and expand public parks and ensure that they are accessible to diverse communities.

Likewise, our study has more general implications for introducing novel policies in times of crisis to influence human behavior. We find that such policies do have a profound effect on how people behave and therefore can be an effective means of mitigating the pandemic (Goldberg *et al.* 2020). Still, our results make evident that there is space for exploration; policies are not final and can be reversed with little adverse effect. We should therefore continue to enact exploratory policies and consider diverse, creative options for dealing with crises such as this. As extreme situations become more frequent with expanding global populations and accelerating land use conversion (Morse *et al.* 2012), understanding how people react to policy decisions will be crucial for planning our responses to future crises.

METHODS A[illegible] MATERIALS

Study area

New Jersey is the United States’ most densely populated state, but, despite this, over 6,070 km^2 of New Jersey are preserved as open space or farmland, totaling approximately 34% of the state’s land area (New Jersey Department of Environmental Protection Green Acres 2018). Our study analyzed the visitation patterns in 98 parks within this substantial park network (Fig. 2). Parks were located in the northern and central portions of the state and span the Piedmont Plains and Skylands ecoregions, which together stretch from the Appalachian Mountains to the Atlantic Ocean and encompass a range of habitats (Michaels *et al.* 1992; Woods *et al.* 2007). Though once widely cultivated, these regions are now largely dominated by successional pine and hardwood woodlands (Collins and Anderson 1994; Woods *et al.* 2007). However, the Piedmont Plains and Skylands ecoregions have been subject to increasing conversion to urban and suburban land cover as a result of New Jersey’s population growth over the past several decades (Woods *et al.* 2007; Wu 2010).

We used the New Jersey Open Space and Preservation Resource Inventory (NJ OSPRI) to identify candidate parks for inclusion in this study (Fig. 2; NJOIT-OGIS 2017). We defined parks broadly, including conservation areas, preserves, recreational facilities (*e.g.* athletic fields), conservation easements, and historic sites. As such, the parks included in this study capture a broad range of recreational uses and fall under a variety of municipality and agency management categories.

Park visitation data

Previous work has demonstrated that social media data can be used to approximate park visitation rates, as the two are highly correlated (Wood *et al.* 2013; Donahue *et al.* 2017; Hamstead *et al.* 2018). We therefore assessed park visitation at our selection of candidate parks using publicly available, geotagged social media data from Instagram (Wood *et al.* 2013; Tenkanen *et al.* 2017). Instagram is an increasingly widely used online social media platform in which users can upload and share photos with their friends or 'followers' (Tenkanen *et al.* 2017; Kats 2018; eMarket editors 2020). Alongside photos, Instagram users can provide captions and georeferenced location tags. Users can make their accounts either private, such that only the user's followers can see their account and posts, or public, such that anyone can see their pictures as well as the photo captions and tags.

To gather data on park usage, we wrote a Python script to collect data on publicly accessible Instagram photos using the Selenium WebDriver package (SFC 2020). We first matched the parks identified above to their corresponding Instagram location tags, of which there were often several for a park, and visually verified that all location tags corresponded to the correct parks using Google maps. After we had created this list of accurate location tags for the parks we identified in NJ OSPRI, we ran our Python script to collect the metadata of all photos associated with each tag; we collected the user handle, date, location tag, photo caption, and hashtags for each photo. We gathered data on photos posted from 2017 to present, as the high volume of photos from some popular parks prohibited the feasibility of collecting pre-2017 data.

After we compiled data on all the photos tagged at these 98 parks, we verified that no photos from parks in other locations were accidentally included in our dataset. This was necessary because parks with shared names but located in other states were occasionally mistakenly included on our park location pages (*e.g.* photos from 'Warren County, Georgia'

ended up on 'Warren County Park, NJ' location page). We also aggregated data for parks that had several different location tags corresponding to different permutations of the park's name (*e.g.* photos tagged 'Delaware and Raritan State Park' and 'D&R Canal State Park' were taken to represent the same location).

Once photos were assigned to the correct parks, we performed two final cleaning steps on our dataset: (1) we removed all photos with 'tbt', 'throwback', and 'flashback' in the tags or comments section, because these are not necessarily reflective of park visitation during the study period; and (2) we subsetted our dataset for parks with data from at least every other week in 2020 (≥11 weeks), to ensure adequate data coverage. Also, due to particular concerns that photos posted during the park shutdown might have been retrospective posts, we went back through photos from the closure period and manually inspected photo comments and tags to remove those that made obvious reference to park trips from before the governor's order.

There were a few limitations to this approach. First, we were only able to use geotagged photos from public accounts using this method, though we can see no obvious reasons this would bias our findings; there is no reason to expect that people with private accounts would post preferentially at certain parks or times. Also, Instagram has a feature called 'Instagram stories' in which users can post photos temporarily (24 hours) for their followers to see (Villaespesa and Wowkowych 2020). Instagram stories have increased in popularity since the feature's introduction in 2016 (Villaespesa and Wowkowych 2020). As stories are transient and not public, our method unfortunately could not capture data from Instagram stories. However, if users increasingly created stories rather than making posts, this would lead us to underestimate park visitation in 2020, thereby underestimating any differences we find between 2020 and previous years. As such, we feel our data are able to accurately capture changes in relative park

visitation, both within and across years (Wood *et al.* 2013; Donahue *et al.* 2017; Tenkanen *et al.* 2017; Hamstead *et al.* 2018).

Altogether, this methodology resulted in a dataset of 160,079 Instagram photos from 98 parks, spanning 2017 to 2020. Of these, 24,807 were from the first five months of 2020 alone.

Other model variables

The beginning of the COVID-19 pandemic was contemporaneous with the onset of spring and resultant changes in weather (Ellis 2020; Korevaar *et al.* 2020; Liu *et al.* 2020). Since the use of public parks and outdoor recreational spaces is highly dependent on the weather, increasing with warmer temperatures and decreasing with precipitation (Richardson and Loomis 2004), we were concerned that the changes in weather associated with the onset of spring might drive our results (Fig. S2a). For instance, park usage would be expected to increase during quarantine simply because of concomitant increases in temperature (Richardson and Loomis 2004). To account for this, we gathered daily temperature and precipitation data for Ewing Township, NJ, which is located in central New Jersey and therefore broadly representative of weather conditions across all parks (Weather Underground 2020). We used weather data to detrend visitation data and compare park usage across time periods when applicable (see *Statistical analyses*). It would have been methodologically impractical to use weather data for each individual park, as we often aggregated data across parks in our analyses (see *Statistical analyses*).

As previously stated, Instagram has increased in popularity over time (Fig. S2a; Tenkanen *et al.* 2017; Kats 2018; eMarket editors 2020). As such, changes in the total number of users – and therefore total number of pictures posted – might drive any observed differences in

park visitation between years. To account for this, we compiled yearly data on the total number of United States Instagram users (Kats 2018; eMarket editors 2020), so we could detrend visitation data and compare park usage across years (see *Statistical analyses*).

Critical to this study was an accurate list of parks that were closed due to the shutdown order, so we could compare trends between closed and open parks. Though the New Jersey governor's order to shut down parks applied primarily to state and county parks (Ellis 2020; Korevaar *et al.* 2020), some other park management agencies followed suit and likewise shut down their parks (Ellis 2020; Korevaar *et al.* 2020). As such, we ascertained which parks were closed and which ones remained open from a variety of sources (see *Supplementary Information* for complete list of sources consulted). Note that for seven out of the 98 parks included here, information on closure status during the executive order was not available (Fig. 2). As such, these seven parks were excluded from all analyses that compared closed and open parks.

Statistical analyses

All data analyses were done in R 3.6.1 (R Core Team 2020). For statistical analyses, we calculated the photograph user days (PUD) as our response variable. PUD is the number of unique combinations of Instagram users and date information for each park (Wood *et al.* 2013; Donahue *et al.* 2017; Hamstead *et al.* 2018). Likewise, for analyses in which we were aggregating across other time scales, not days, we used the number of unique Instagram users for that time period. In both cases we did this to avoid inflation in our visitation metrics from individuals who uploaded several photos of the same park on the same day and to prevent any photos that may have been included under several different permutations of a park's location tag from being double (or triple) counted.

Role of public parks in the pandemic

To assess whether the onset of the pandemic and the stay-at-home order were associated with increased park visitation, we compared park usage during the first month of the pandemic (period 2) to park visitation during the same time period in previous years (with the date range adjusted slightly to have the same number of weekdays and weekends). We first detrended the PUD data using annual estimates of the number of monthly active US Instagram users to account for increased popularity of the platform over time (Fig. S2; Tenkanen *et al.* 2017; Kats 2018; eMarket editors 2020). To the accommodate the non-normality of the data and the negative non-integer detrended PUD values, we calculated bootstrapped estimates of model coefficients for all our analyses. For this research question, we modeled detrended PUD, aggregated across parks, as a function of year, maximum daily temperature, and daily precipitation rate (Fig. 3a). We considered a model variable to have an effect on park visitation (*i.e.* 'be significant') if the 95% confidence interval did not overlap zero.

To further determine whether the work from home order was associated with increased park visitation, we compared PUD during the first month of quarantine (period 2) to the equivalent time frame before COVID-19 quarantine restrictions were put in place in 2020 (period 1). For this analysis, we did not accommodate the change in Instagram user numbers, as Instagram user data were only available at annual scale. We used our bootstrap methodology to model PUD, aggregated across parks, as a function of pre- and post-quarantine status, maximum daily temperature, and daily precipitation rate (Fig. 3b).

Efficacy of the executive order

To determine whether the New Jersey governor's park closure order was associated with changes in park usage, we compared visitation patterns before (period 2) and during (period 3) the order in parks that were made to close and parks that remained open (Fig. 4). However, closed parks vastly outnumbered open parks ($n_{closed} = 79$, $n_{open} = 12$). To account for this, we simulated 1000 datasets with equivalent numbers of open and closed parks ($n = 12$) and calculated bootstrap estimates of the effect of park closure for each dataset. Furthermore, the parks that closed had substantially higher historical visitation rates than parks that remained open, as they were the more widely used county and state parks (Fig. S2b). Therefore, we evaluated the percent change in visitation levels from before and after the executive order, rather than absolute visitation, and how this related to park status (open vs. closed). Temperature was not included as a variable in this analysis since it did not differ between the two time periods (periods 2 and 3) according to a separate bootstrap analysis.

Lifting of the executive order

To determine whether reopening parks was associated with changes in park visitation patterns, we first compared park usage during May 2020, following the withdrawal of the executive order (period 4), to visitation during the same interval in previous years (Fig. 5a). We calculated bootstrap estimates of the effect of year, maximum temperature, and daily precipitation on detrended PUD, aggregated across parks.

Next, we determined how visitation changed in parks after lifting the park closure order in 2020 (comparing periods 3 and 4). We again wanted to assess whether patterns differed between formerly closed parks and parks that had remained open, so we simulated 1000 datasets with equivalent numbers of closed and open parks. For this analysis we detrended PUD by

temperature since temperatures in March/early April differed from those in May. Then we generated our bootstrap estimates of executive order status (during vs. after park closure) and its interaction with park status (open vs. closed) on our detrended PUD measure (Fig. 5b).

Finally, we wanted to determine how post-order visitation levels (period 4) compared to park visitation during the initial stages of the pandemic (period 2), to see whether park usage increased in the aftermath of the park shutdown, particularly in the parks that had been closed. Again, we detrended PUD by temperature (since temperature differed between the two periods). For this analysis, we subsetted our post-executive order dataset (period 4) to only the first four weeks of May to have an equal number of days and weekends between the two periods. Then we generated our bootstrap estimates of executive order status (before vs. after park closures) and its interaction with park status (open vs. closed) on our detrended PUD measure (Fig. 5c).

STATEMENT OF AUTHORSHIP: ZMV and APD designed the study; ZMV collated and analyzed the data; ADB contributed to analysis design and manuscript development. JOA led the manuscript development and writing with significant contributions from ZMV. All authors contributed manuscript feedback.

LITERATURE CITED

Anguelovski, I., (2020). Expanding the boundaries of justice in urban greening scholarship: Towards an emancipatory, antisubordination, intersectional, and relational approach. *Annals of the American Association of Geographers* 1–59.

Buckley, R., Brough, P., Hague, L., Chauvenet, A., Fleming, C., Roche, E., ... & Harris, N. (2019). Economic value of protected areas via visitor mental health. *Nature communications*, 10(1), 1-10.

CDC (2020). Guidance for Administrators in Parks and Recreational Facilities. Available at: *https://www.cdc.gov/coronavirus/2019-ncov/community/parks-rec/park-administrators.html*

Collins, B.R., & Anderson, K.H. (1994). *Plant Communities of New Jersey*. Rutgers University Press, New Brunswick, NJ.

Deforche, K., Vercauteren, J., Müller, V., & Vandamme, A. M. (2020). Behavioral changes before lockdown, and decreased retail and recreation mobility during lockdown, contributed most to the successful control of the COVID-19 epidemic in 35 Western countries. *medRxiv*.

Donahue, M. L., Keeler, B. L., Wood, S. A., Fisher, D. M., Hamstead, Z. A., & McPhearson, T. (2018). Using social media to understand drivers of urban park visitation in the Twin Cities, MN. *Landscape and Urban Planning*, *175*, 1-10.

Du, J., King, R., Chanchani, R., 2020. Tackling Inequality in Cities is Essential for Fighting COVID-19. *World Resources Institute*.

Ellis, N.T. (2020). These national and state parks are closed due to the coronavirus outbreak. *USA Today.*

eMarketer editors (2020). The Digital World in 2020. *eMarketer.com.* Available at: *https://www.emarketer.com/content/podcast-the-digital-world-in-2020?ecid=NL1001*

Fortuna, L. R., Tolou-Shams, M., Robles-Ramamurthy, B., & Porche, M. V. (2020). Inequity and the disproportionate impact of COVID-19 on communities of color in the United States: The need for a trauma-informed social justice response. *Psychological Trauma: Theory, Research, Practice, and Policy.*

Freeman, S., & Eykelbosh, A. (2020). COVID-19 and outdoor safety: Considerations for use of outdoor recreational spaces. *National Collaborating Centre for Environmental Health*.

Godbey, G., Graefe, A. R., & James, S. W. (1992). *The benefits of local recreation and park services: a nationwide study of the perceptions of the American public*. Arlington, VA: National Recreation and Park Association.

Goldberg, M. H., Gustafson, A., Maibach, E. W., Ballew, M. T., Bergquist, P., Kotcher, J. E., ... & Leiserowitz, A. (2020). Mask-wearing increased after a government recommendation: A natural experiment in the US during the COVID-19 pandemic. *Frontiers in Communication*, *5*, 44.

Hamstead, Z. A., Fisher, D., Ilieva, R. T., Wood, S. A., McPhearson, T., & Kremer, P. (2018). Geolocated social media as a rapid indicator of park visitation and equitable park access. *Computers, Environment and Urban Systems*, *72*, 38-50.

Haynes, N., Cooper, L. A., Albert, M. A., & Association of Black Cardiologists. (2020). At the Heart of the Matter: Unmasking and Addressing the Toll of COVID-19 on Diverse Populations. *Circulation*, 142(2), 105-107.

Higgins-Dunn, N. and Kim, J. (2020). New Jersey Gov. Phil Murphy reopens state parks and golf courses. *CNBC.*

Honey-Roses, J., Anguelovski, I., Bohigas, J., Chireh, V., Daher, C., Konijnendijk, C., ... & Oscilowicz, E. (2020). The impact of COVID-19 on public space: a review of the emerging questions.

Jacobson, N. C., Lekkas, D., Price, G., Heinz, M. V., Song, M., O'Malley, A. J., & Barr, P. J. (2020). Flattening the Mental Health Curve: COVID-19 Stay-at-Home Orders Are Associated With Alterations in Mental Health Search Behavior in the United States. *JMIR mental health*, *7*(6), e19347.

Jennings, V., Johnson Gaither, C., & Gragg, R. S. (2012). Promoting environmental justice through urban green space access: A synopsis. *Environmental Justice*, 5(1), 1-7.

Kats, R. (2018). Who Is Using Instagram in the US? *eMarketer.com.* Available at: *https://www.emarketer.com/content/the-social-series-who-s-using-instagram*

Korevaar, H. M., Becker, A. D., Miller, I. F., Grenfell, B. T., Metcalf, C. J. E., & Mina, M. J. (2020). Quantifying the impact of US state non-pharmaceutical interventions on COVID-19 transmission. *medRxiv*.

Liu, P., Beeler, P., & Chakrabarty, R. K. (2020). COVID-19 Progression Timeline and Effectiveness of Response-to-Spread Interventions across the United States. *medRxiv*.

Matias, T., Dominski, F. H., & Marks, D. F. (2020). Human needs in COVID-19 isolation. *Journal of Health Psychology,* 25(7), 871–882.

Michaels, J.A., Neville, L.R., Edelman, D., Sullivan, T., DiCola, L.A. (1992). *New York-New Jersey Highlands regional study.* USDA Forest Service, Northeastern Area-State and Private Forestry, Syracuse, NY.

Manning, R., & More, T. (2002). Recreational values of public parks. *The George Wright Forum* 19(2):21-30. George Wright Society.

Morse, S. S., Mazet, J. A., Woolhouse, M., Parrish, C. R., Carroll, D., Karesh, W. B., Zambrana-Torrelio, C., Lipkin, W. I., Daszak, P. (2012). Prediction and prevention of the next pandemic zoonosis. *Lancet* 380 (9857), 1956–1965.

New Jersey Department of Environmental Protection Green Acres. (2018). *2018-2022 New Jersey Statewide Comprehensive Outdoor Recreation Plan*. Available at: *https://www.nj.gov/dep/greenacres/pdf/2018_draft_SCORP.pdf.*

New Jersey Office of Information Technology, Office of Geographic Information Systems (NJOIT-OGIS). (2017). *Open Space and Preservation Resources Inventory (OSPRI).* NJ DEP, Trenton, NJ.

New York Times (2020). *New Jersey Coronavirus Map and Case Count.* [cited August 17th, 2020]. Available at: *https://www.nytimes.com/interactive/2020/us/new-jersey-coronavirus-cases.html*

Nutsford, D., Pearson, A. L., & Kingham, S. (2013). An ecological study investigating the association between access to urban green space and mental health. *Public health*, *127*(11), 1005-1011.

R Core Team (2020). *R: A language and environment for statistical computing*. R Foundation for Statistical Computing, Vienna, Austria. Documentation at: *https://www.R-project.org/.*

Richardson, R. B., & Loomis, J. B. (2004). Adaptive recreation planning and climate change: a contingent visitation approach. *Ecological Economics*, 50(1-2), 83-99.

Rundle, A. G., Park, Y., Herbstman, J. B., Kinsey, E. W., & Wang, Y. C. (2020). COVID- 19–Related School Closings and Risk of Weight Gain Among Children. *Obesity*.

Samuelsson, K., Barthel, S., Colding, J., Vetenskapsakademien, K., Giusti, M., (2020). Urban nature as a source of resilience during social distancing amidst the coronavirus pandemic. Research Gate Pre-print. doi:10.31219/osf.io/3wx5a.

SFC (2020). Selenium WebDriver. *Software Freedom Conservancy (SFC).* Documentation at: *https://www.selenium.dev/documentation/en/webdriver/*

Tenkanen, H., Di Minin, E., Heikinheimo, V., Hausmann, A., Herbst, M., Kajala, L., & Toivonen, T. (2017). Instagram, Flickr, or Twitter: Assessing the usability of social media data for visitor monitoring in protected areas. *Scientific reports*, *7*(1), 1-11.

Torales, J., O'Higgins, M., Castaldelli-Maia, J. M., & Ventriglio, A. (2020). The outbreak of COVID-19 coronavirus and its impact on global mental health. *International Journal of Social Psychiatry*, 0020764020915212.

Ulrich, R. S., & Addoms, D. L. (1981). Psychological and recreational benefits of a residential park. *Journal of Leisure Research*, *13*(1), 43-65.

Valentino-DeVries, Lu, D., Dance, G.J.X., (2020). *Location Data Says It All: Staying at Home During Coronavirus Is a Luxury.* The New York Times.

Venter, Z., Barton, D., Figari, H., & Nowell, M. (2020). Urban nature in a time of crisis: recreational use of green space increases during the COVID-19 outbreak in Oslo, Norway.

Villaespesa, E., & Wowkowych, S. (2020). Ephemeral Storytelling With Social Media: Snapchat and Instagram Stories at the Brooklyn Museum. *Social Media & Society* 6(1): 2056305119898776.

Viner, R. M., Russell, S. J., Croker, H., Packer, J., Ward, J., Stansfield, C., ... & Booy, R. (2020). School closure and management practices during coronavirus outbreaks including COVID-19: a rapid systematic review. *The Lancet Child & Adolescent Health*.

Weather Underground. (2020). Ewing Township, NJ Weather History. [cited July 22nd, 2020]. Available at: *https://www.wunderground.com/history/daily/us/nj/montgomery-township/KTTN*

Wolch, J. R., Byrne, J., & Newell, J. P. (2014). Urban green space, public health, and environmental justice: The challenge of making cities 'just green enough'. *Landscape and urban planning*, 125, 234-244.

Wood, S. A., Guerry, A. D., Silver, J. M., & Lacayo, M. (2013). Using social media to quantify nature-based tourism and recreation. *Scientific reports*, *3*(1), 1-7.

Wood, L., Hooper, P., Foster, S., & Bull, F. (2017). Public green spaces and positive mental health–investigating the relationship between access, quantity and types of parks and mental wellbeing. *Health & place*, *48*, 63-71.

Woods, A. J., Omernik, J. M., & Moran, B. C. (2007). *Level III and IV ecoregions of New Jersey.* US Environmental Protection Agency, National Health and Environmental Effects Research Laboratory, Corvallis, OR, 1(9).

Wu, S. (2010). New Jersey Population: 1790-2010. *New Jersey Economic Indicators* 531: 19-21.

FIGURES

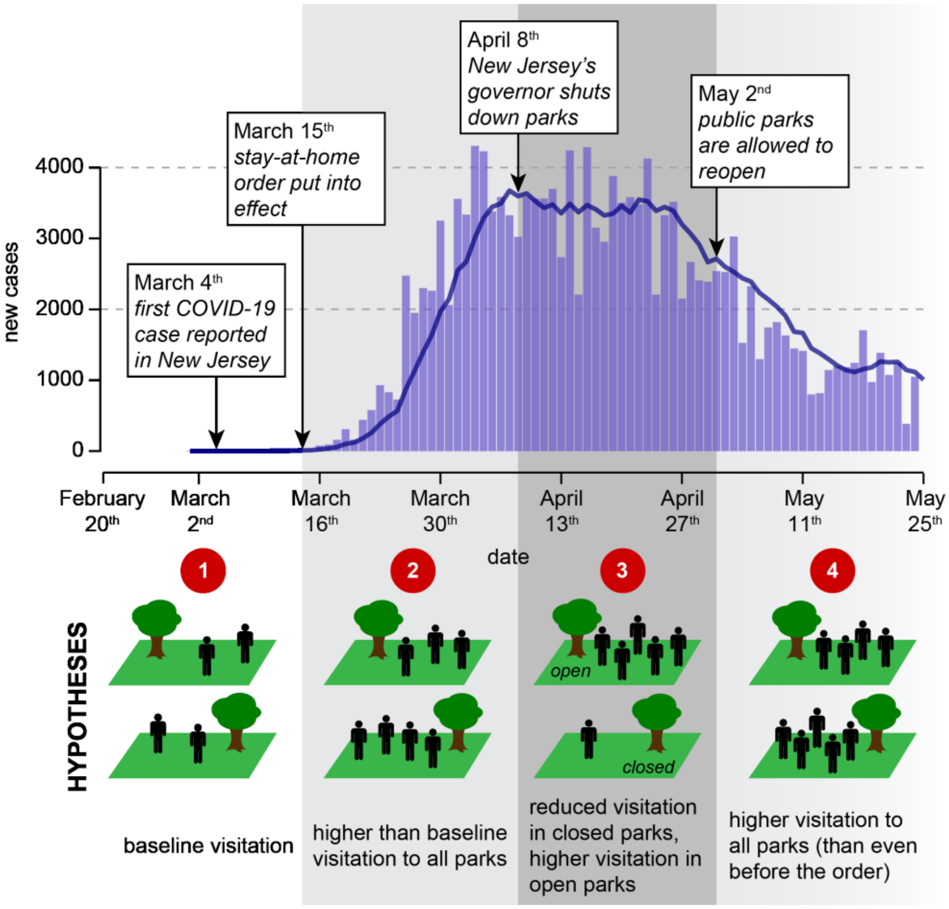

Figure 1. Daily new COVID-19 cases reported in New Jersey, with key events marked and their predicted impacts on the use of public parks depicted. Light purple bars correspond to the number of new cases per day, whereas the dark purple line reflects the number of new cases averaged over the previous seven days. We predict that the stay-at-home order (period 2) caused people to use parks more (relative to baseline usage before quarantine; period 1). We predict that the subsequent park shutdown order (period 3) was largely effective at reducing park usage in closed parks but possibly concentrated people in the parks that remained open. Finally, we predict that the end of the shutdown order (period 4) caused people to return to parks as they had before the shutdown. New Jersey COVID-19 case data are from The New York Times (2020).

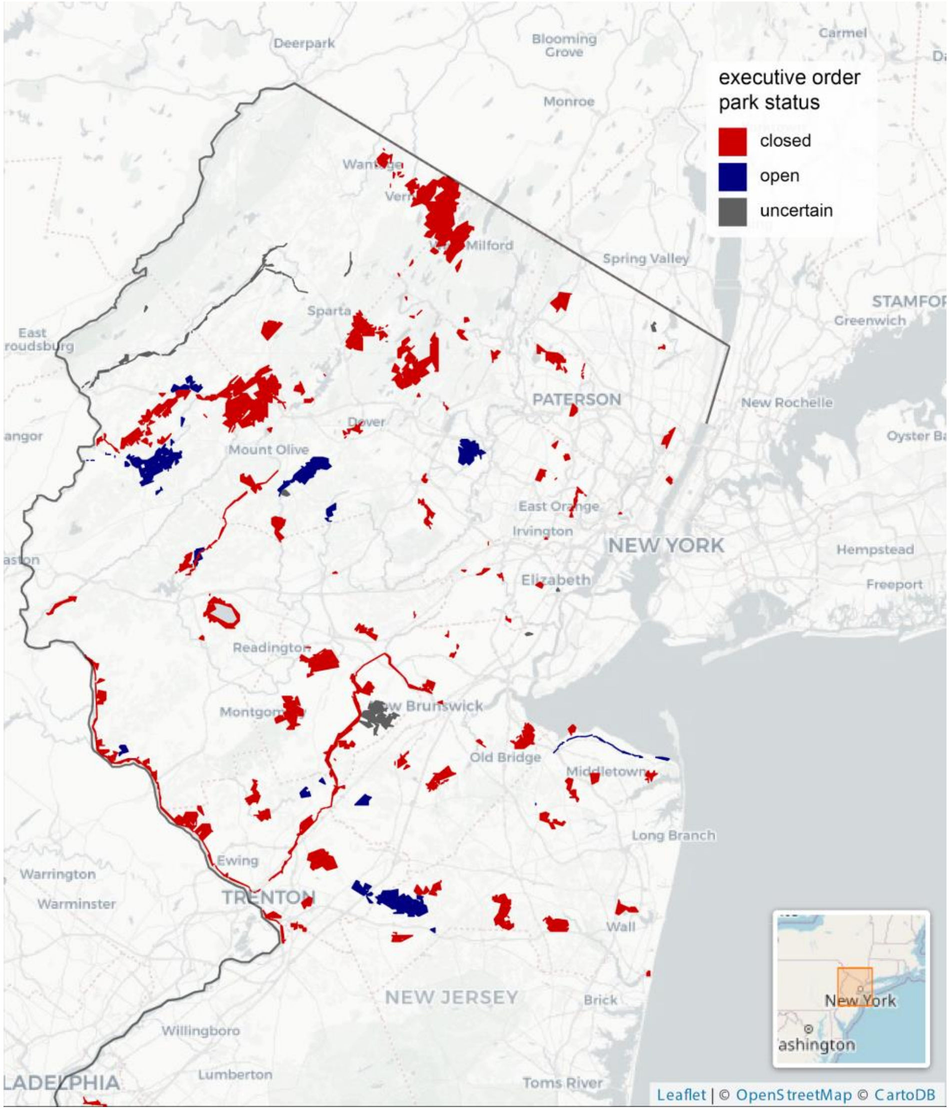

Figure 2. The distribution of New Jersey parks included in this study (*n* = 98). Parks in blue (*n* = 12) remained open through the whole study period, whereas parks in red (*n* = 79) were closed during the governor's executive order (April 8th - May 2nd). For parks colored in grey (*n* = 7), we were uncertain whether they were open or closed during the park shutdown, so these parks were excluded from all analyses comparing closed and open parks.

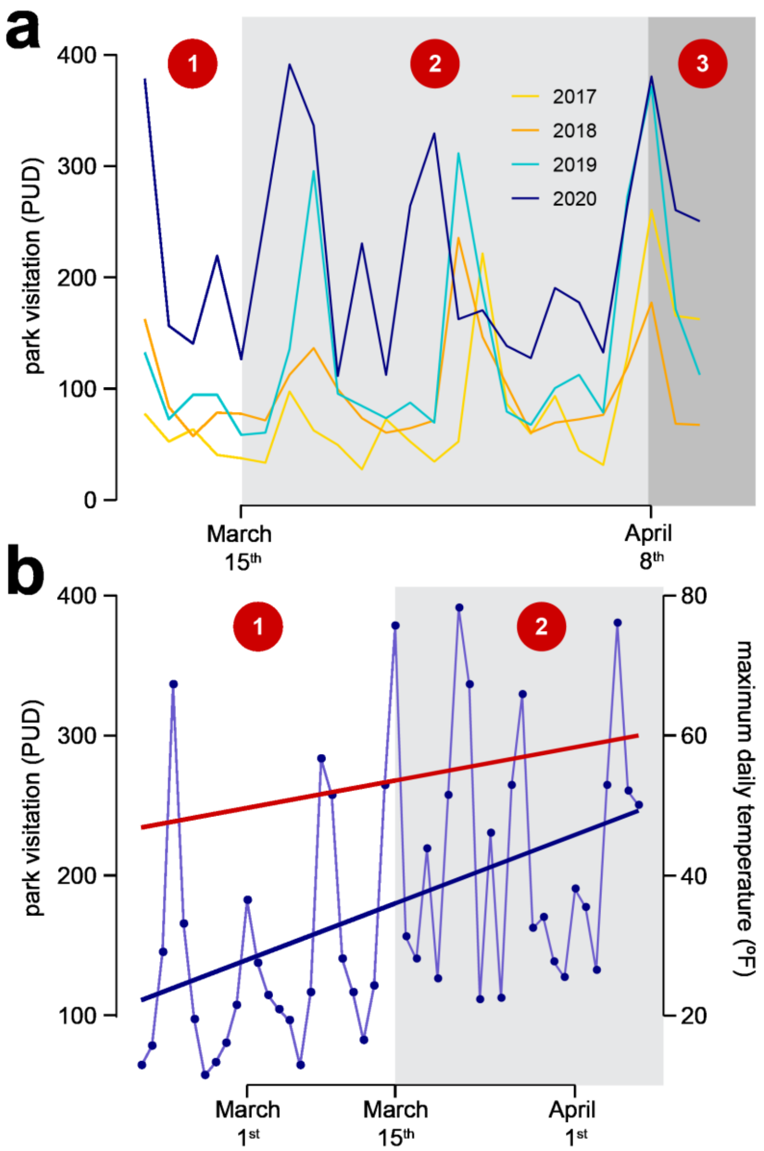

Figure 3. The effects of the onset of the COVID-19 pandemic on park visitation (as measured by PUD, photograph user days). Park usage during mid-March through early April of 2020 was higher relative to previous years (a), and park visitation increased when the work from home order was put into effect, even after controlling for temperature increases (b). The numbers in red circles refer to the time periods denoted in Fig. 1.

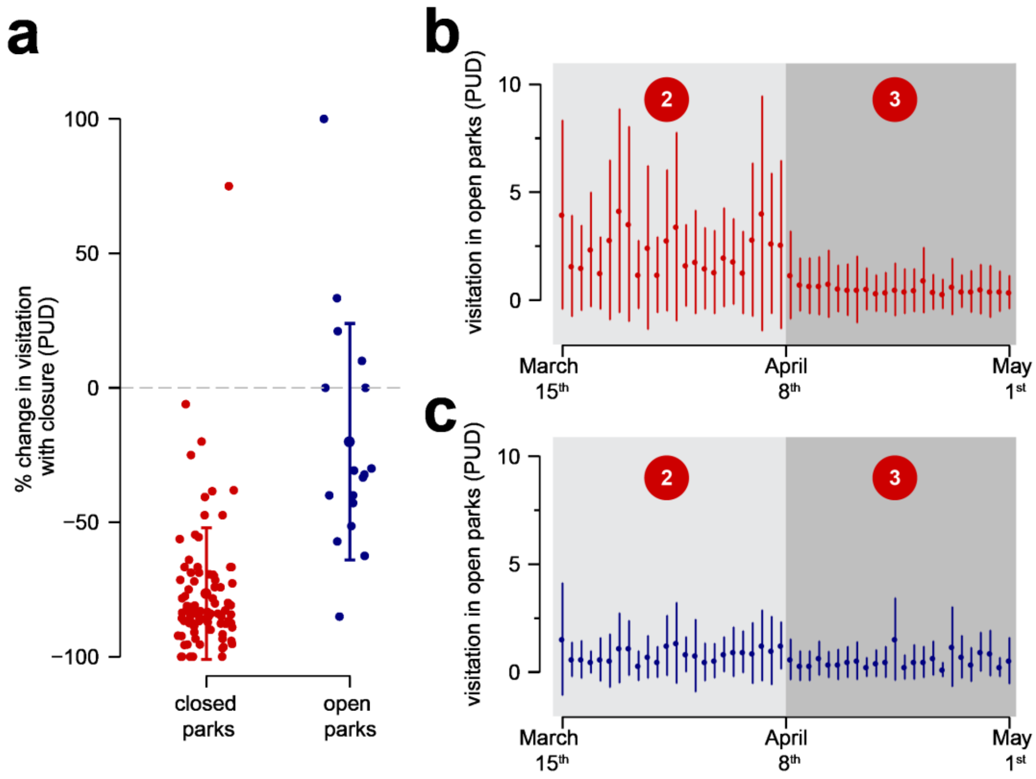

Figure 4. The impact of the New Jersey governor's executive order to shut down parks on park visitation (a). Visitation in closed parks dropped substantially (b), whereas changes in visitation to parks that remained open were much more variable (c). The numbers in red circles in (b) and (c) refer to time periods denoted in Fig. 1.

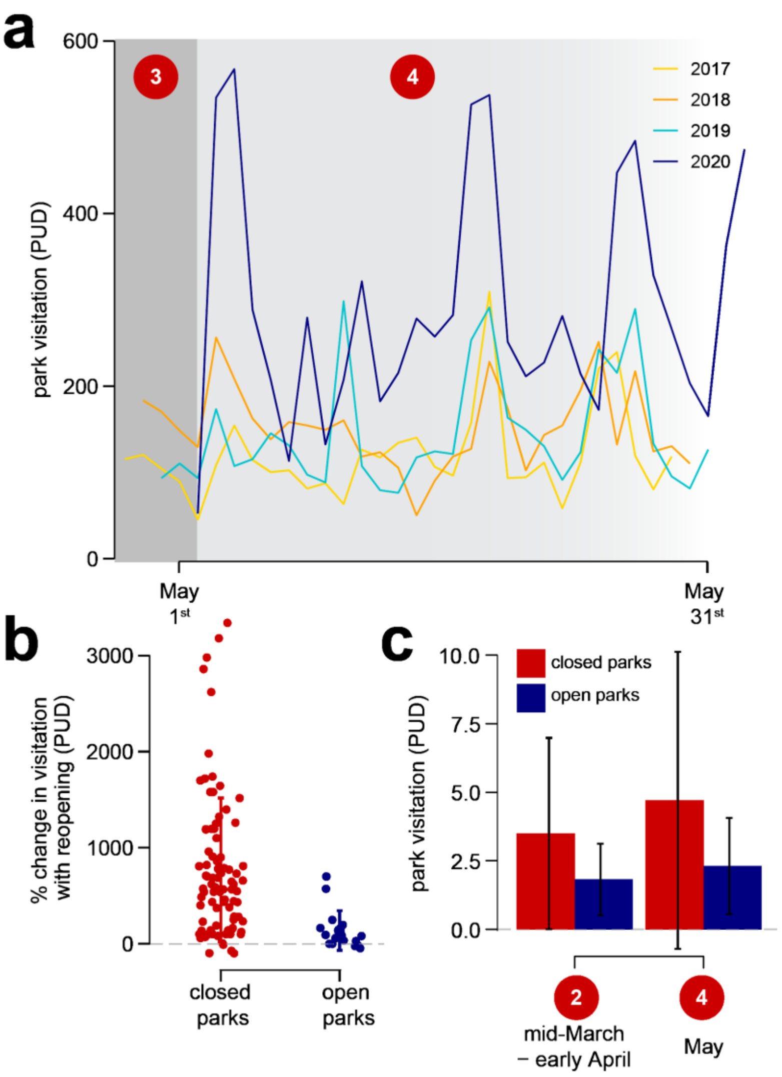

Figure 5. The effects of the lifting of the park shutdown order on park visitation. Park usage during May 2020 was higher relative to previous years (a). Increases in visitation were much larger in formerly closed parks as compared to parks that had remained open (b). However, visitation did not exceed visitation levels from before the shutdown (c). The numbers in red circles in (a) and (c) refer to time periods denoted in Fig. 1.

SUPPLEMENTARY INFORMATION

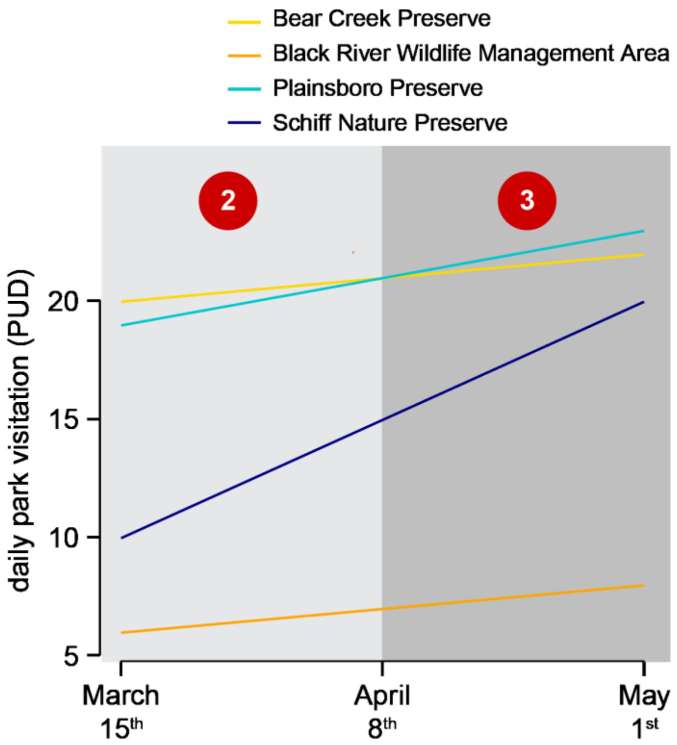

Figure S1. Changes in visitation (as measured by PUD) in the four open parks where visitation increased after the governor's executive order. Visitation to Schiff Nature Preserve almost doubled, whereas increases were much more moderate in Bear Creek Preserve, Black River Wildlife Management Area, and Plainsboro Preserve. The numbers in red circles refer to the time periods denoted in Fig. 1.

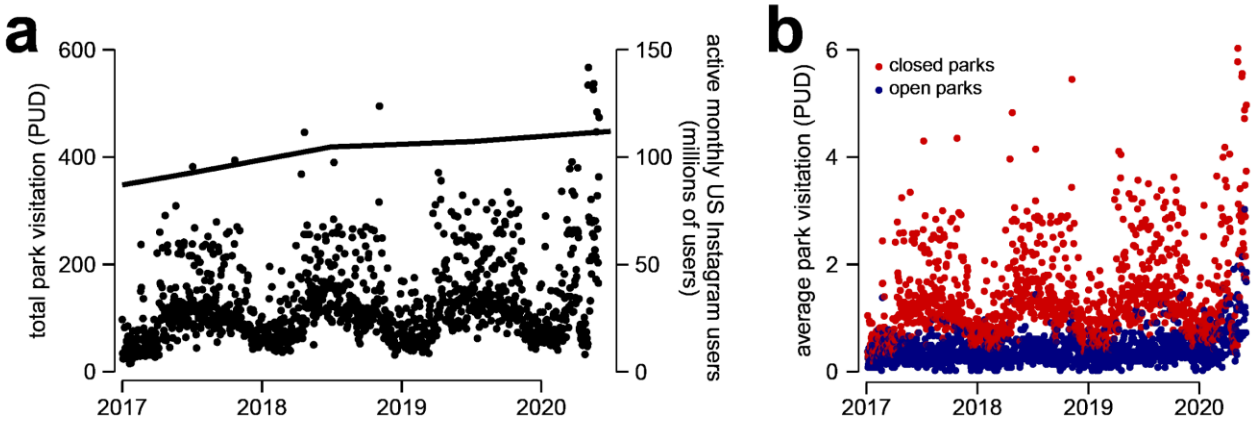

Figure S2. Total park visitation (PUD summed across all parks) during the study period (2017-2020; a) as well as average visitation for parks that were closed or open during the executive order (b). The black line in (a) reflects the trend in the number of active US Instagram users through time, which was used to detrend PUD values when comparing park usage across years. Note in (a) that Instagram usage has increased through time, and also the distinct seasonality of park visitation. Likewise, note in (b) that parks that were closed during the park shutdown consistently had higher visitation historically than parks that remained open.

List of sources consulted to determine park closure status during the shutdown

Friends of Hopewell Valley Open Space. (2020). *COVID-19 Updates.* [cited April 18th, 2020]. Available at: *https://www.fohvos.info/covid-19-updates/*

Hunterdon County Division of Parks and Recreation. (2020). *Your County Parks*. [cited April 18th, 2020]. Available at: *http://www.co.hunterdon.nj.us/depts/parks/guides/parkareas.htm*

New Jersey Department of Environmental Protection. (2020). *Open spaces at state parks and wildlife management areas available for passive recreation; enclosed facilities, campgrounds at state parks, forests and recreation areas closed*. [cited April 18th, 2020]. Available at: *https://www.nj.gov/dep/newsrel/2020/20_0010.htm*

NJ.com. (2020). *What N.J. parks are closed? A full list of parks Gov. Murphy just closed in response to coronavirus.* [cited April 18th, 2020]. Available at: *https://www.nj.com/coronavirus/2020/04/is-my-local-park-closed-a-full-list-of-parks-gov-murphy-just-closed-in-response-to-coronavirus.html*

Planet Princeton. (2020). *Open and closed: Princeton area parks.* [cited April 18th, 2020]. Available at: *https://planetprinceton.com/2020/04/08/open-and-closed-princeton-area-parks/*

Somerset County Parks Division. (2020). *COVID-19 Press Release.* [cited April 18th, 2020]. Available at: *https://www.somersetcountyparks.org/COVID-19%20Press%20Release%209-2.pdf*

Tewksbury Township. (2020). *COVID-19 Resources.* [cited April 18th, 2020]. Available at: *https://www.tewksburytwp.net/coronavirus_(covid-19)/coronavirus_(covid-19).php*

Union County. (2020). *Parks, Activities, Locations & Maps.* [cited April 18th, 2020]. Available at: *https://ucnj.org/parks-activities/*

Village Green NJ. (2020). *Essex County Closes All Parks, Playgrounds, Dog Parks to Prevent Spread of COVID-19.* [cited April 18th, 2020]. Available at: *https://villagegreennj.com/towns/government/march-16-2020-essex-county-announces-additional-precautions-to-prevent-spread-of-covid-19/*
